# Analysis of a Larger SNP Dataset from the HapMap Project Confirmed That the Modern Human A Allele of the ABO Blood Group Genes Is a Descendant of a Recombinant between B and O Alleles

**DOI:** 10.1155/2013/406209

**Published:** 2013-10-29

**Authors:** Masaya Itou, Mitsuharu Sato, Takashi Kitano

**Affiliations:** ^1^Department of Biomolecular Functional Engineering, College of Engineering, Ibaraki University, 4-12-1 Nakanarusawa-cho, Hitachi 316-8511, Japan; ^2^Department of Medical Genome Science, Graduate School of Frontier Sciences, The University of Tokyo, 5-1-5 Kashiwanoha, Kashiwa 277-8561, Japan

## Abstract

The human ABO blood group gene consists of three main alleles (A, B, and O) that encode a glycosyltransferase. The A and B alleles differ by two critical amino acids in exon 7, and the major O allele has a single nucleotide deletion (Δ261) in exon 6. Previous evolutionary studies have revealed that the A allele is the most ancient, B allele diverged from the A allele with two critical amino acid substitutions in exon 7, and the major O allele diverged from the A allele with Δ261 in exon 6. However, a recent phylogenetic network analysis study showed that the A allele of humans emerged through a recombination between the B and O alleles. In the previous study, a restricted dataset from only two populations was used. In this study, therefore, we used a large single nucleotide polymorphism (SNP) dataset from the HapMap Project. The results indicated that the A101-A201-O09 haplogroup was a recombinant lineage between the B and O haplotypes, containing the intact exon 6 from the B allele and the two critical A type sites in exon 7 from the major O allele. Its recombination point was assumed to be located just behind Δ261 in exon 6.

## 1. Introduction

The human ABO blood group consists of three major types, A, B, and O [1]. These alleles code for glycosyltransferases, with the terminal sugar chain modifications varying between types. The enzyme encoded by functional alleles of type A and B transfer a GalNac or a Gal on the precursor oligosaccharides of type H. The nucleotide sequences of the human ABO blood group genes have been previously determined and the molecular basis of these differences has been revealed [2, 3]. The alleles A and B differ in exon 7 by four nonsynonymous mutations, and two of which are critical for the sugar specificity (codons 266 and 268 encode L-G for A and M-A for B). The major O allele has a single nucleotide deletion (Δ261) in exon 6 [4] that induces a frameshift, resulting in a truncated protein deprived of any glycosyltransferase activity.

Major haplogroups (A101, A201, B101, O01, O02, and O09) exist in the human ABO blood group genes [5, 6]. A101 and B101 are the main haplogroups for the A and B alleles, respectively. The activity of A201 is reduced 20- to 50-fold compared to A101, because A201 has a point deletion at nucleotide position 1061 that results in a frameshift adding 21 additional amino acid residues to the protein [7]. O01, O02, and O09 are the main haplogroups of the O type. A series of nucleotide differences have been observed between O01 and O02 [5, 6, 8]. Although O09 shares Δ261 with O01 and O02, its sequence is quite similar to A101. Thus, O09 most likely evolved from an ancestral A101-like common allele by a gene conversion in exon 6, introducing Δ261 from another O allele [5, 6].

Several studies have examined the evolution of the human ABO blood group genes [5, 8–11]. These studies have identified that the A allele is the most ancient, because the chimpanzee, which is the closest relative of humans, has A and O alleles. The O allele of the chimpanzee has evolved by a different mechanism compared to humans [10, 12]. The B allele diverged from the A allele, with nucleotide substitutions on the two critical residues in exon 7. The O02 allele diverged from the A allele with a single nucleotide deletion (Δ261) in exon 6, after which the O01 allele diverged from the O02 allele. In contrast, there are some studies [13, 14] that argue transspecies polymorphism of the A and B alleles. In any case, these studies suggested that these alleles have been maintained by balancing selection.

Recently, a new model for the human ABO blood group genes has been developed, using phylogenetic network analysis [6]. They argued that although the B and O alleles diverged from the A allele, the modern human A allele is not a direct descendant of the ancestral A allele. The modern human A allele emerged through a recombination between the B and O alleles, where the intact exon 6 from the B allele and two critical A type sites in exon 7 from the O allele were jointed less than 300,000 years ago. Since the previous study [6] used a restricted dataset, that is, Seattle SNPs Project data, which is a set of 90 sequences in European- and African-Americans, it is necessary to analyze a more comprehensive dataset to corroborate this hypothesis. Therefore, in the present study, we used SNP data from the HapMap Project to examine the evolution of the human ABO blood group genes.

## 2. Materials and Methods

### 2.1. Datasets

We retrieved two kinds of phased haplotype datasets for the ABO blood group genes from the HapMap Project [15]. The first was the three population dataset (3pop_data) that included the Yoruba in Ibadan, Nigeria (YRI), CEPH (Utah residents with ancestry from northern and western Europe) (CEU), Japanese in Tokyo, Japan, and Han Chinese in Beijing, China (JPT + CHB) (HapMap Data Rel 24/phaseII Nov08, on NCBI B36 assembly, dbSNP b126). The other dataset contained eleven populations (11pop_data) including African ancestry in Southwest USA (ASW), Utah residents with Northern and Western European ancestry from the CEPH collection (CEU), Han Chinese in Beijing, China (CHB), Chinese in Metropolitan Denver, Colorado (CHD), Gujarati Indians in Houston, Texas (GIH), Japanese in Tokyo, Japan (JPT), Luhya in Webuye, Kenya (LWK), Mexican ancestry in Los Angeles, California (MEX), Maasai in Kinyawa, Kenya (MKK), Toscans in Italy (TSI), and Yoruba in Ibadan, Nigeria (YRI) (HapMap Data PhaseIII/Rel#3, May 10, on NCBI B36 assembly, dbSNP b126).

Since haplotypes from most populations in the HapMap Project are estimated from genotypes, there is a possibility that the data might contain erroneous haplotypes. To reduce the possibility of artificial recombinants, we prepared two kinds of datasets from 3pop_data and 11pop_data. The datasets of 3pop_data_1 and 11pop_data_1 consisted of haplotypes from homozygous individuals and individuals carrying only one heterozygous site. Meanwhile, the 3pop_data_2 and 11pop_data_2 datasets consisted of haplotypes observed more than two times from the all populations.

### 2.2. Phylogenetic Analysis

Phylogenetic networks were constructed manually following the procedures of [9, 16]. The chimpanzee sequence data (NW_003457497) was used as an outgroup.

### 2.3. Detection of Recombinant

**Figure 1:**
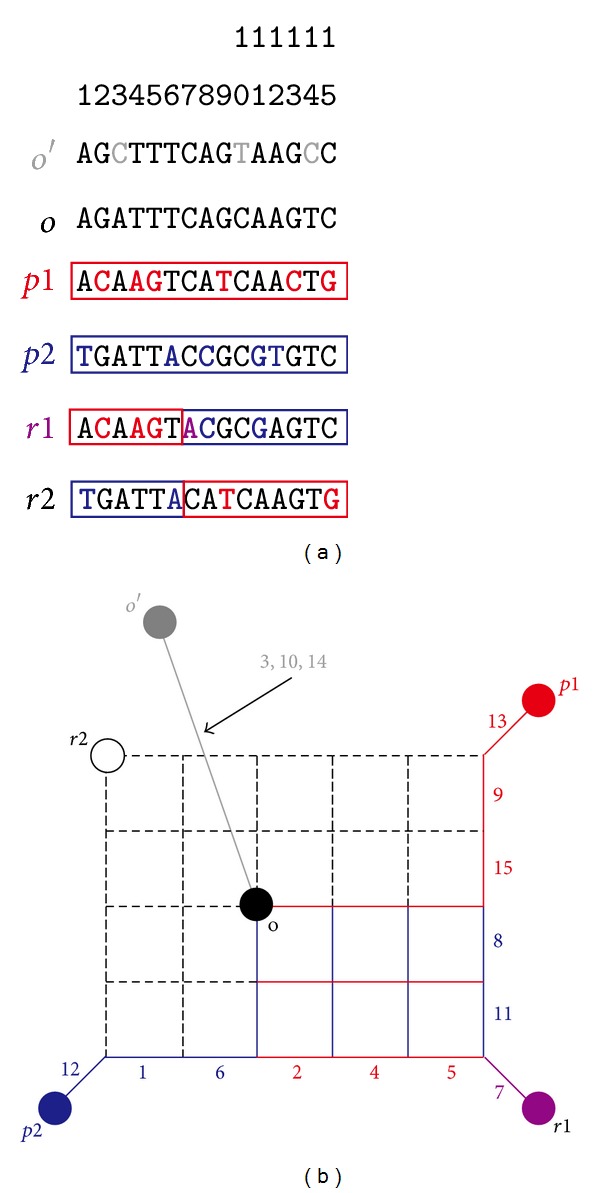
Explanation of a recombination event in a phylogenetic network using model data. A nucleotide sequence (a) and a corresponding phylogenetic network (b) are shown. Modified from [6]. See text for the details.

We attempted to detect recombinants from the phylogenetic network analysis following the procedure of [17]. They showed the relationship between a recombinant and its two parental alleles in a phylogenetic network. We used model data to explain how to infer a recombination event from a phylogenetic network (Figure 1). First, an ancestry sequence (o) produces two different sequences (p1 and p2) (Figure 1(a)). The p1 has five substitutions at sites 2, 4, 5, 9, and 15 (bold red), and the p2 has four substitutions at sites 1, 6, 8, and 11 (bold blue), from sequence o. Then, if recombination occurred between sites 6 and 7 for p1 and p2, two recombinants (r1 and r2) exist. After the recombination, three nucleotide substitutions at sites 7 (purple), 12 (blue), and 13 (red) accumulate to p1, p2, and r1, respectively, and three nucleotide substitutions at sites 3, 10, and 14 (gray) also accumulate to produce an outgroup (o′) from sequence o. Assuming that r1 and r2 were produced by a single recombination event, transmission of both recombinant alleles to the next generation is highly improbable. Therefore, we assumed that r2 had disappeared.  Figure 1(b) is the phylogenetic network represented by Figure 1(a) at the time. The phylogenetic network (Figure 1(b)) indicates the relationship between the extant alleles (p1, p2, and r1) and an outgroup (o′). Two parental alleles (p1 and p2) are located on opposing vertices of the rectangle and have longer (compared to that of the recombinant allele) external branches (sites 13, 9, and 15 for p1 and sites 12, 1, and 6 for p2), while the recombinant allele (r1) is located on the vertex opposing the outgroup allele (o′) and has a shorter (compared to those of parental alleles) external branch (site 7). “External branch” means a single line extended from reticulations to an external node here.

The PNarec (phylogenetic network-based recombination detection) method [18], a general application of [17], was applied for selected haplotypes (they are representative haplotypes from haplogroups) using the PNarec program (available from Supplementary Material of [18]).

## 3. Results and Discussion

### 3.1. Phylogenetic Analysis of the Human ABO Blood Group Genes

**Figure 2:**
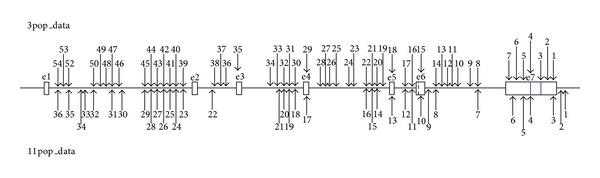
The location of SNP sites on the human ABO blood group genes obtained by the HapMap Project. A dashed line indicates Δ261, and blue vertical lines indicate the two critical sites that determine the blood group A and B specificities. The SNP number is shown in the Supplementary Material.

Fifty-four and 36 SNPs for 3pop_data and 11pop_data, respectively, were retrieved from the HapMap Project (Figure 2). We predicted ABO types for each haplotype (see Supplementary Material available online at http://dx.doi.org/10.1155/2013/406209) by using haplotype-specific SNPs, following data of [5]. Since insertion and deletion variations are not contained in the data, A201 and O09 could not be distinguished from A101. These haplogroups are treated as the A101-A201-O09 haplogroup, because it is highly likely that A201 and O09 evolved from A101 [5, 6].

**Figure 3:**
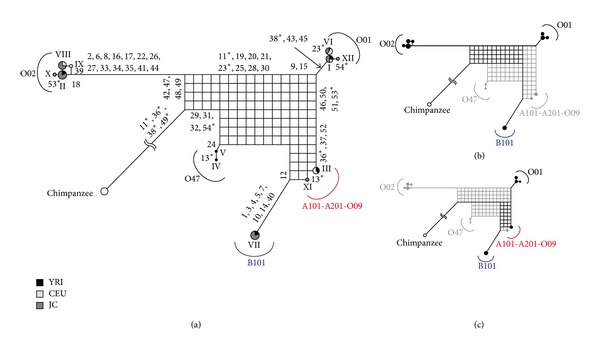
The phylogenetic network constructed from 3pop_data_1 (a). A circle graph presented on each node indicates the proportion of three populations, and the size of the circle graph indicates frequency of each haplotype. The numbers on each branch are nucleotide positions responsible for those branches. The underlined haplotypes were used for the PNarec program. The reticulation of the phylogenetic network for a recombinant and its parental haplogroups is shown by thick lines ((b) and (c)).

Figure 3(a) describes a phylogenetic network of the human ABO blood group genes using 3pop_data_1. Since the O01 haplogroup has a shorter external branch and is located on the vertex opposing the outgroup, the O01 haplogroup is thought to be a recombinant lineage. Its parental allele lineages are expected to be the O02 and B101 haplogroups, because the O02 and B101 haplogroups are located on opposing vertices of the rectangle with longer external branches (Figure 3(b)). Twelve sites (11, 19, 20, 21, 23, 25, 28, 29, 30, 31, 32, and 54) support O01 and B101 haplogroup clustering, while four sites (42, 47, 48, and 49) support O01 and O02 haplogroup clustering. Thus, the recombination point is estimated to be between sites 32 and 42. This corresponds to a region between intron 1 and intron 3 (Figure 2). Substitutions at site 54 might be parallel ones. Recombination events detected in the study are summarized in Table 1. If we cannot expect to have a recombination event, we need to assume four (42, 47, 48, and 49) parallel substitutions at the O01 and O02 lineages or 12 (11, 19, 20, 21, 23, 25, 28, 29, 30, 31, 32, and 54) parallel substitutions at the O01 and B101 lineages. In the phylogenetic network, the A101-A201-O09 haplogroup is also thought to be a recombinant lineage, and its parental lineages are the O01 and B101 haplogroups (Figure 3(c)). Three sites (9, 13, and 15) support A101-A201-O09 and O01 haplogroup clustering, while seven sites (36, 37, 46, 50, 51, 52, and 53) support A101-A202-O09 and B101 haplogroup clustering. Thus, the recombination point is estimated to be between sites 15 and 36, which correspond to a region between intron 2 and exon 6. Since A101 and A201 do not have Δ261, we can expect that the recombination point is located in exon 6 (between sites 15 and Δ261). If we do not assume a recombination event, we should assume three parallel substitutions (9, 13, and 15) at the A101-A201-O09 and O01 lineages or seven parallel substitutions (36, 37, 46, 50, 51, 52, and 53) at the A101-A201-O09 and B101 lineages. The O47 haplogroup is thought to be a recombinant between O47 and some other haplogroups [5]. However, in the phylogenetic network, the O47 haplogroup is not located on the vertex opposing the outgroup, probably because its parental O47 haplogroup lineage is not included in the data.

**Figure 4:**
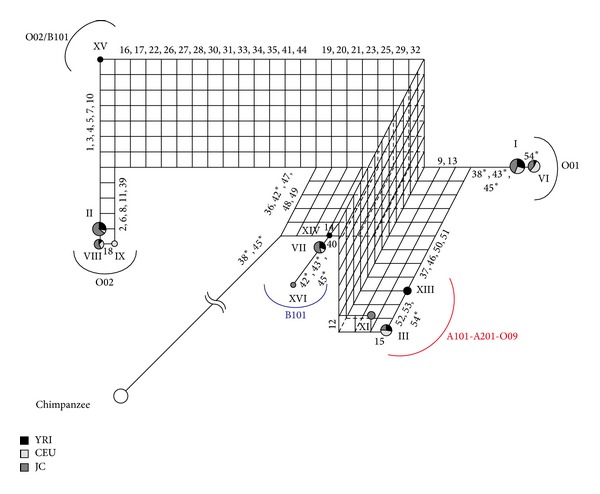
The phylogenetic network constructed from 3pop_data_2. Details are as in Figure 3.

**Figure 5:**
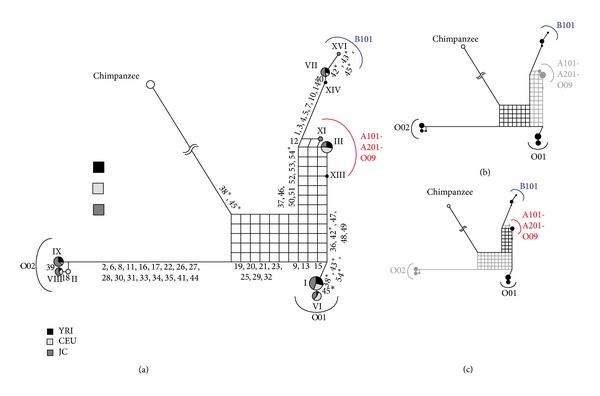
The phylogenetic network constructed from 3pop_data_2, except for the XV (O02/B101) haplotype. Details are as in Figure 3.

Figure 4 describes a phylogenetic network using 3pop_data_2. The haplotype XV is thought to be a recombinant between the O02 and B101 haplogroups [5, 6]. Thus, we designated this as O02/B101. Since this haplotype has Δ261 [5], the recombination point should be located between Δ261 and site 12 (Table 1). Thus, in contrast with other O type alleles which have two critical A type sites in exon 7, this O type allele has two critical B type sites. The O02/B101 haplotype is observed only in African populations (YRI of HapMap data and African Americans of the Seattle SNPs data). We reconstructed a phylogenetic network excluding the haplotype O02/B101 (Figure 5(a)), which shows similar results compared to Figure 3. The O01 haplogroup is thought to be a recombinant lineage, where the O02 and B101 haplogroups are its parental allele lineages (Figure 5(b)). Seven sites (19, 20, 21, 23, 25, 29, and 32) support O01 and B101 haplogroup clustering, while five sites (36, 42, 47, 48, and 49) support O01 and O02 haplogroup clustering. Thus, the recombination point is estimated to be between sites 32 and 36. This corresponds to a region between intron 2 and intron 3 (Figure 2). It is also thought that the A101-A201-O09 haplogroup is a recombinant lineage, where its parental lineages are the O01 and B101 haplogroups (Figure 5(c)). We can expect that the recombination point is located in exon 6 (between sites 32 and Δ261), because A101 and A201 should not have Δ261. If we do not assume a recombination event, we should assume three parallel substitutions (9, 13, and 15) at the A101-A201-O09 and O01 lineages or seven parallel substitutions (37, 46, 50, 51, 52, 53, and 54) at the A101-A201-O09 and B101 lineages. These results strongly support the hypothesis that the modern human A allele is derived from a recombination event between the O01 and B101 lineages [6].

**Figure 6:**
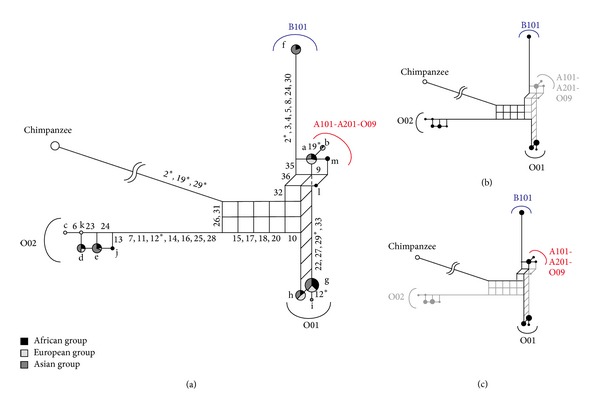
The phylogenetic network constructed from 11pop_data_1. Details are as in Figure 3.

We also used 11pop_data, which contains data from 993 individuals from the world, but the number of SNPs is lower at 36 compared to 54 in 3pop_data.  Figure 6(a) describes a phylogenetic network using 11pop_data_1. This phylogenetic network indicated similar results to 3pop_data; the O01 and A101-A201-O09 haplogroups are recombinant lineages (Figures 6(b) and 6(c)). In addition, since each haplogroup consists of individuals from several populations, it is suggested that they had been formed before divergences of human populations, that is, prior to migration out of Africa.

**Figure 7:**
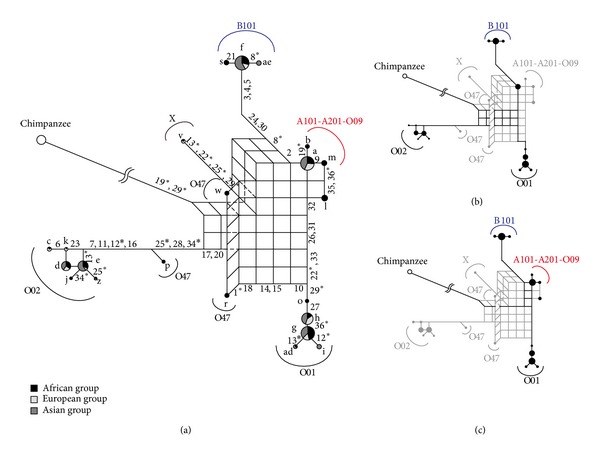
The phylogenetic network constructed from 11pop_data_2. Details are as in Figure 3.

Figure 7(a) describes a phylogenetic network using 11pop_data_2. This phylogenetic network indicated similar results to the above phylogenetic networks. Some minor haplotypes (Table 2), which probably occurred by recombination or gene conversion, were not included in the phylogenetic network to prevent construction of a complex multidimensional phylogenetic network. This phylogenetic network showed similar results with the above phylogenetic networks; the O01 and A101-A201-O09 haplogroups are recombinant lineages (Figures 7(b) and 7(c)). Three O47 haplotypes (r, p, and w) were separated from each other in the phylogenetic network. This result is not unexpected, because O47 haplotypes are thought to be recombinants between O47 and some other haplogroups [5], and these three haplotypes (r, p, and w) share only one O47 specific site (site 1 in Figure 2; see also Supplementary Material). In addition, we observed the v haplotype, which is a rare haplotype. The v haplotype was assigned to the haplogroup X tentatively, because this haplotype could not be predicted as a known haplogroup (see Supplementary Material).

### 3.2. Application of the PNarec Program

We applied the PNarec program using some selected haplotypes. We used five haplotypes (I, II, III, V, and VII) from 3pop_data_1, five haplotypes (I, II, III, VII, and XV) from 3pop_data_2, and four (a, e, f, and g) from 11pop_data_1, as representative haplotypes. Representative haplotypes from 11pop_data_2 were the same as those from 11pop_data_1. The results are also summarized in Table 1. It is suggested that the A101-A201-O09 haplogroup is derived from a recombination event in all the datasets, though O02 is assigned as a parental allele instead of O01. The PNarec method [18] is composed of five steps (A~E). Step A: count the numbers of singleton sites for each sequence, Step B: choose all possible quartet sequences, including the outgroup and count the three types of phylogenetically informative sites, Step C: choose quartets in which one of the splits had no phylogenetically informative site, Step D: examine the distribution of the remaining two phylogenetically informative sites for each quartet, and choose ones whose site distributions do not overlap each other, and Step E: choose the quartet whose recombinant descendant lineage sequence candidate has the smallest number of singleton sites. If there is more than one candidate with the smallest number of singleton sites, choose the quartet in which the two parental descendant lineage sequence candidates have the largest sum of singleton sites. Until Step D, O01 remains as a candidate of a parental allele. At Step E, O02 is chosen as a parental allele instead of O01, because O02 has a longer external branch than O01. The PNarec method is still under development [18], and further improvements to this the method are required.

The PNarec program did not detect O01 as a recombinant from all four datasets. O01 was eliminated from recombinant candidates at Step C, because O01 has parallel changes with the chimpanzee outgroup. Meanwhile, it was suggested that O01 appears to resemble a mosaic of B101 and O02 by gene conversions rather than simple recombination [6]. Thus, O01 might not be a simple recombinant. The haplotype XV is clearly detected as a recombinant between the O02 and B101 haplogroups.

### 3.3. Evolutionary History of the Human ABO Blood Group Genes

**Figure 8:**
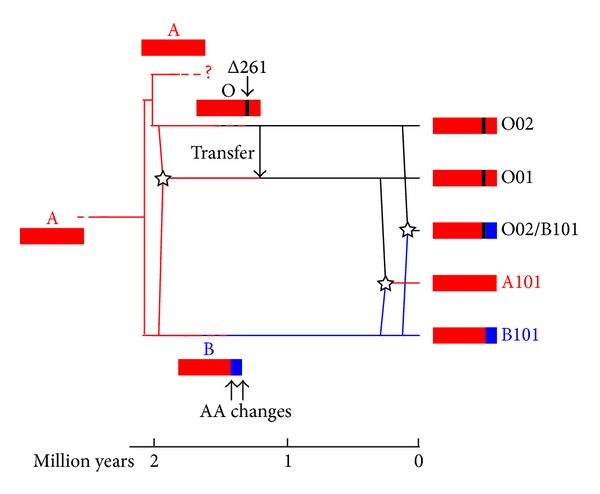
A scheme of the evolutionary pathway of some haplogroups of the human ABO blood group genes. Red square indicates the A type gene. Black vertical bar indicates Δ261 in exon 6. The blue portion of the B gene indicates the two B type-specific critical amino acid sites; containing region in exon 7. Stars indicate recombination event. A rough time scale is shown by following the results of [6]; the divergence time of the lineage leading to B101: 2.08 million years ago, the divergence time of the lineage leading to O01: 1.98 million years ago, and the divergence time of the lineage leading to A101: 0.26 million years ago. Because times of divergence between A and O02, the Δ261 deletion, transfer of Δ261, two amino acid substitutions for B101, and recombination for O02/B101 cannot be estimated, these positions are located arbitrarily.

Figure 8 depicts a possible evolutionary scheme of haplogroups for the human ABO blood genes. We assume A as an ancestor in humans, because chimpanzees mainly have A alleles [10, 12, 19]. B101 then diverged from A, followed by substitutions for the two critical sites, and O (O02) diverged from A with Δ261. O01 might be formed by a recombination coupled with gene conversions (including transfer of Δ261) between B101 and O02 [6]. The O02/B101 haplotype was formed by a recombination between O02 and B101.

A101 is the recombinant product with the intact exon 6 from B101 and two critical A type sites in exon 7 from O01 that had been joined to form the functional A allele. The results from this study, using the HapMap data, mirror the results of [6]. A101 should be distinct from the ancestral A allele, which produced the B101 and O02 haplogroups. It is not clear whether the ancestral A allele coexisted with other haplogroups in modern humans. If the ancestral A allele exists in the human population, it should be located near the common ancestral position with a longer external branch in a phylogenetic network. We observed the v haplotype (X haplogroup), which is located near the common ancestral position in a phylogenetic network (Figure 7). Since this haplotype consists of individuals from ASW, CEU, GIH, MEX, and TSI, it seems to have been formed prior to migration out of Africa. Thus, it is possible to expect that the X haplogroup may be the ancestral A haplotype, that is, the most ancient haplotype. Further studies are needed to clarify whether the X haplogroup is indeed the ancestral A allele.

## Supplementary Material

Single nucleotide polymorphism datasets used in the study.

## Figures and Tables

**Table 1:** Recombinants expected from phylogenetic networks and PNarec.

Data	Recombinant	Parent 1 (forward)	Parent 2 (backward)	Region of recombination point
3pop_data_1	O01	O02	B101	32 and 42
	A101-A201-O09*	B101	O01 (O02)	15 and Δ261 (15 and 36)
3pop_data_2	XV* (O02/B101)	O02	B101	12 and Δ261 (12 and 16)
	O01	O02	B101	32 and 36
	A101-A201-O09*	B101	O01	15 and Δ261 (15 and 36)
11pop_data_1	O01	O02	B101	20 and 26
	A101-A201-O09*	B101	O01 (O02)	10 and Δ261 (1 and 11)
11pop_data_2	O01	O02	B101	20 and 26
	A101-A201-O09*	B101	O01 (O02)	10 and Δ261 (1 and 11)

Recombinants estimated not only from a phylogenetic network but also from the PNarec method are indicated by asterisks. Notation in parentheses indicates the result expected from PNarec.

**Table 2:** Minor haplotypes which were not included in the phylogenetic network constructed by 11pop_data_2.

Haplotype (possible haplogroup)	Number of sequences	Estimated cause (recombination point and its parental haplotypes*)
n (A101-A201-O09/O47)	5	Recombination event (b, 22–26, r)
q (O02/O47)	11	Recombination event (e, 26–28, w)
t (B101/A101-A201-O09)	6	Recombination event (f, 19–24, a/b/m)
u (B101/A101-A201-O09)	5	Recombination event (f, 2-3, a)
x (O01/A101-A201-O09)	4	Recombination event (g/h, 22–26, a/b/m)
y (B101/O02)	13	Recombination event (f, 7-8, e)
aa (O01/O02)	5	Recombination event (g/h, 25–27, c/d/e)
ab (B101 with O01)	4	Gene conversion (26–29 of g/h/i was converted into f)
ac (A101-A201-O09-O01/O47)	6	Recombination event and one nucleotide substitution (a/b/g/h, 10–14, r, and 1 substitution)
af (B101)	3	Recombination event (a/b/c/d/e/g/h/i/j/k/l/m, 2-3, f)
ag (O02/A101-A201-O09)	7	Recombination event (d/e, 20–23, a/b/m)
ai (A101-A201-O09-O01/O02)	7	Recombination event (a/b/g/h, 13-14, j)
ah (O47/B101)	7	Recombination event and one nucleotide substitution (p/r/w, 8–14, f, and 1 substitution)

*For example, haplotype q is a recombinant between e (forward) and w (backward) between sites 26 and 28.
